# Mitochondrial Membrane Studies Using Impedance Spectroscopy with Parallel pH Monitoring

**DOI:** 10.1371/journal.pone.0101793

**Published:** 2014-07-10

**Authors:** Divya Padmaraj, Rohit Pande, John H. Miller, Jarek Wosik, Wanda Zagozdzon-Wosik

**Affiliations:** 1 Electrical and Computer Engineering Department, University of Houston, Houston, Texas, United States of America; 2 Texas Center for Superconductivity, University of Houston, Houston, Texas, United States of America; 3 Physics Department, University of Houston, Houston, Texas, United States of America; University of Iowa, United States of America

## Abstract

A biological microelectromechanical system (BioMEMS) device was designed to study complementary mitochondrial parameters important in mitochondrial dysfunction studies. Mitochondrial dysfunction has been linked to many diseases, including diabetes, obesity, heart failure and aging, as these organelles play a critical role in energy generation, cell signaling and apoptosis. The synthesis of ATP is driven by the electrical potential across the inner mitochondrial membrane and by the pH difference due to proton flux across it. We have developed a tool to study the ionic activity of the mitochondria in parallel with dielectric measurements (impedance spectroscopy) to gain a better understanding of the properties of the mitochondrial membrane. This BioMEMS chip includes: 1) electrodes for impedance studies of mitochondria designed as two- and four-probe structures for optimized operation over a wide frequency range and 2) ion-sensitive field effect transistors for proton studies of the electron transport chain and for possible monitoring other ions such as sodium, potassium and calcium. We have used uncouplers to depolarize the mitochondrial membrane and disrupt the ionic balance. Dielectric spectroscopy responded with a corresponding increase in impedance values pointing at changes in mitochondrial membrane potential. An electrical model was used to describe mitochondrial sample’s complex impedance frequency dependencies and the contribution of the membrane to overall impedance changes. The results prove that dielectric spectroscopy can be used as a tool for membrane potential studies. It can be concluded that studies of the electrochemical parameters associated with mitochondrial bioenergetics may render significant information on various abnormalities attributable to these organelles.

## Introduction

Mitochondria are very complex organelles that perform a number of vital cellular functions. Their primary role is energy conversion, which results in production of adenosine triphosphate (ATP), the primary source (over 90%) of energy for cells. Besides ATP generation, mitochondria also perform other cell-specialized tasks that differ in functionality depending on the tissue or organism. Mitochondria are vital for cell life and death (they initiate apoptosis), cell signaling, modulation of intracellular calcium ion fluxes and mediation of cell protection. Moreover, mitochondria adapt to varying energy requirements, which change in response to energy consumption, and control production of free radicals called reactive oxygen species (ROS) identified as the main cause of aging and of various serious and degenerative diseases. Hence mitochondria are vital in our physiology, and understanding their structure and operation has pathological importance in studying human life and health. Mitochondrial functioning relies on complex ion transfer processes happening within various protein complexes present at the inner membrane. Characterization of these processes is difficult, partly also because of mitochondria isolation ambiguity and partly because of existing constraints on realization of selective and multi-parametric testing methods.

According to Mitchell’s chemiosmotic theory [1], in the electron transport chain (ETC) a proton motive force (Δp) develops at the inner mitochondrial membrane (IMM), which includes predominant membrane potential (ΔΨ_m_) and the pH gradient between matrix and cytosol (ΔpH). The ATP synthase protein complex within the IMM couples the proton-transport back across the inner membrane into the matrix to produce ATP. The membrane potential ΔΨ_m_ ≈120 mV [2] both optimizes ATP production and minimizes the generation of free radicals. In unstressed living cells, ΔΨ_m_ values are in the range of 100–130 mV [3] but increase to about 200 mV under stress and drop below 100 mV when permeability transition pores (PTP) open. Too little membrane polarization with low concentration of protons results in insufficient ATP production, whereas a large membrane potential (ΔΨ_m_>150 mV) leads to excessive generation of superoxide free radicals (ROS) and therefore contributes to various diseases and to aging [4]. Moreover, changes in the process kinetics within ETC complexes due to varying contributions of ΔΨ_m_ and ΔpH affect Δp, which further complicates not only the monitoring of mitochondria physiological normal functions but also dysfunctions identified in various diseases [5]. About 40 diseases [6], [7] including age-related diseases have been linked to defects in mitochondrial function. That includes various cancer types, where mitochondria play a major role whereby cancer cells undergo uncontrolled proliferation and growth via alteration of their metabolism from oxidative phosphorylation to glycolysis, known originally as Wartburg effect [8], [9].

Thus the potential across the IMM is a key indicator of cell viability and mitochondrial activity. It is a highly sensitive indicator of the energetic state of mitochondria and health of cells [7], [10], and can be used to investigate the activity of the proton pump and electron transport system as well as the state of mitochondrial permeability [11]. ΔΨ_m_ is usually determined by fluorescent probing or patch clamp methods. Fluorescent dyes show noise limitations in measurements and modification of the membrane potential by the penetrating and accumulating dye, causing measurement inaccuracy [12].

We used a noninvasive impedance spectroscopy technique for mitochondrial membrane studies. For ionic activity study of mitochondria we added ion-sensitive field effect transistors (ISFETs) to measure pH changes. To test the efficacy of this tool we used uncouplers, which may disrupt the ionic balance and depolarize the mitochondrial membrane. Such a tool can act as a sensor for detection and studies of mitochondrial dysfunction linked to the membrane. Dielectric spectroscopy, upon addition of uncoupler, showed an increase of impedance due to changes in mitochondrial membrane potential that varied with frequency.

## Materials and Methods

### Ethics statement

The experimental protocol and program care has been reviewed and approved by the University of Houston Institutional Animal Care and Use Committee (protocol 10-027 “Electromagnetic Harmonic Spectroscopy as Sensors of Metabolic Activity”). Each animal employed for tissue harvest mammalian mitochondria for the coupled/uncoupled experiments was subjected to high standards of care. For mitochondrion extraction we have used isoflurane for anesthetizing mice and directly after tissue harvest mice were decapitated.

### 1. Materials

**Figure 1:**
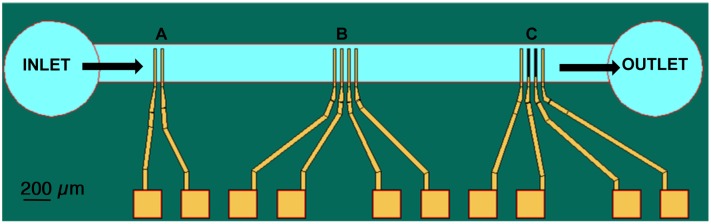
Schematic of the fabricated three electrode arrays with two electrodes (a), four electrodes (b) and four electrodes mesh (c) is shown here with the microfluidic channel across the active part of the electrodes.

A biological microelectromechanical system (BioMEMS) was fabricated using traditional microelectronic processes, but included modifications related to specific materials used. Two and four-electrode arrays were designed for impedance spectroscopy. The electrodes are 50 µm wide and 600 µm long, with 100 µm spacing between them (Fig. 1). Four-electrode arrays have the advantage of reduced electrode polarization effects. As the current is supplied across the outer electrodes, and the inner electrodes are only voltage pick up electrodes, current flow through the inner sensing electrodes is negligible. Such a feature makes the four-electrode configuration more preferable for impedance measurements than the two-electrode configuration. The electrodes were fabricated by thermal evaporation of gold at a 0.5-µm thickness, with chromium as the adhesion layer, on a silicon substrate with a thermally grown silicon dioxide layer. Subsequent patterning was done using optical lithography in the contact printing mode and positive photoresist.

Microfluidic channels with a height of 50 µm were fabricated by patterning SU-8 resist to control fluid flow to each electrode. The whole wafer was then mounted on a printed circuit board and wire-bonded to make electrical contacts. For measurement comparison, a two-electrode array with the same dimensions was also incorporated (Fig. 1). We have also included a similar array using mesh electrodes, in which the two inner electrodes consisted of a mesh with square holes sized 7.5 µm by 7.5 µm patterned in the Cr/Au layer. This was done to reduce polarization effects due to double layer formation between the electrode and solution at low frequency [13].

For mitochondria measurements we used the following buffers: buffer A: 40.08 g 220 mM mannitol (MW = 182.17)+23.96 g 70 mM sucrose (MW = 342.3)+1.05 g 5 mM Mops (MW = 209.3) in 1 liter of water; buffer B: 0.076 g 2 mM EGTA (MW = 380.4)+4 ml of 5%FAF BSA for 0.2% BSA in 100 ml of buffer A; and buffer C: 0.019 g 0.5 mM EGTA in 100 ml buffer A. The measurements were done on freshly pelleted out mice cardiac mitochondria extracted using a typical extraction protocol: The mouse was isofluoraned for 15 seconds. Heart was extracted, weighed and placed in glass test tube on ice. Afterwards the heart was washed four times with buffer A and then minced on ice. Minced heart was put in 6 ml of buffer B and then polytron treatment at low for 60 seconds. Centrifuged for 600 g for 10 min at 4°C and then the supernatant was saved. The pellet was resuspended in 4 ml of buffer A and then centrifuged 600 g for 10 min at 4°. Supernatant was combined and then centrifuged again at 3000 g for 15 minutes at 4°. Finally the pellet was resuspended in buffer C.

Stripping the outer membrane or compromising the inner membrane must be avoided, since that would lead to uncoupled mitochondria, in which the inner membrane contains proton leaks so that electron transport is not coupled to ATP synthesis. Based on the Biuret method, the mitochondrial sample concentration was determined to be 4 mg/ml using a spectrophotometer. These isolated mitochondria are devoid of any source of energy and do not consume oxygen because of the deficit of fuel in the form of fatty acids or substrates. To activate respiration in the ETC, starting with complex I, we added glutamate and malate as substrates. These substrates are consumed in the citric acid cycle to generate reduced nicotinamide adenine dinucleotide NADH, which donates electrons to complex I. The energy from these electrons is used by the ETC to build up the proton gradient and membrane potential across the IMM. To probe dielectric properties of the samples, which correlate with changes in membrane potential [14], we used the protonophore FCCP (carbonyl cyanide 4-(trifluoromethoxy) phenylhydrazone). FCCP depolarizes the IMM and collapses the membrane potential by permeating the membrane and allowing free passage of protons across it [15]. It permeates both the inner and the outer membrane. The drop in membrane potential is expected to cause a drop in low-frequency dielectric response [14] and corresponding increase in impedance.

**Figure 2:**
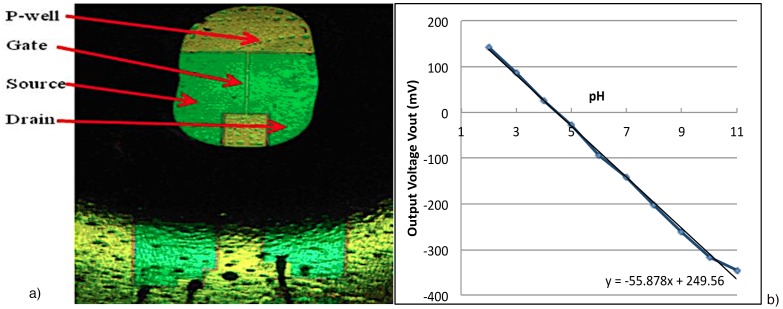
Structure of the fabricated ISFET [16] as optical microscope picture (a) and ISFET response to different pH solutions (b) are shown.

For complementary measurements of pH, we used Ion Sensitive Field Effect Transistors, which respond to accumulation of electrical charges on the ion-sensitive gate dielectric from the investigated samples. Changes of ionic concentations in the solution modify threshold voltage of ISFET and its current therefore allow for ion sensing. These transistors were fabricated on (100) n-type (500 Ω*-*cm) Si substrates using basic complementary metal oxide semiconductor (CMOS) technology [16]. The channel width and length of the ISFET structures were 600 µm and 16 µm, respectively. Silicon nitride deposited on thermal oxide was used as a gate dielectric and acted as an ion-sensitive membrane. Source/drain leads were fabricated to provide enough separation between the pads and active area exposed to bio-electrolyte [16], [17] as shown in Figure 2a. The ISFETs were encapsulated in a resin, exposing only the silicon nitride gate region.

The interfacing electronics for the ISFET measurements is a very significant attribute for obtaining reliable and stable results. The constant drain-source voltage and constant drain-source current mode−based interface circuit, which we use here, allows for stable ISFET response [17]. With ISFETs, there are many sources of noise, including external electromagnetic fields and solution perturbance. Changes of pH in the solution affect the threshold voltage therefore changes drain current and the output voltage. To improve system stability, a reference voltage generating circuit is added. This circuit [17] employs a Zener diode, whose one side is connected to ground. An operational floating amplifier−based circuit is used to provide a floating bias driver to the sensing circuit. The circuit employs a bridge-type sensing circuit with two embedded low pass filters. These filters improve the signal-to-noise ratio.

ISFETs were calibrated at room temperature; the operating point was fixed at a drain-source voltage of 0.5 V and a drain current of 100 µA. The output voltages were noted down for different buffer solutions. Buffer solutions ranging from pH 2 to 11 were prepared and used for the pH calibration of ISFET by noting down the respective output voltage at the source for the particular pH buffer (Fig. 2b). Here the ISFET displayed a sensitivity of 55.878 mV/pH. The calibration curve shows a good linear relationship between the output voltage and pH.

### 2. Equivalent circuit modeling

The basic methodology of an impedance characterization experiment involves three steps: 1) Electrical measurements are done to characterize contents of the unknown sample; the data obtained are used to describe the electrical behavior and hence explain the physical and chemical processes going on inside; 2) The behavior should be based on a model, which can be an equivalent circuit, which mimics the electrical behavior of the sample; 3) The equivalent model obtained is a tool to interpret the results. Here we discuss the development of an electrical equivalent circuit for our mitochondrial sample. Only by developing a model, we can analyze the individual features and dependencies of the system under study.

The mitochondrion consists of a double-membraned cylindrical structure enclosing the mitochondrial matrix and the inter membrane space. Hence the important features of the structure are the membranes and the fluidic spaces enclosed by them. The intracellular impedance of cells is usually represented by an ohmic resistance to an axial flow of current [18]. The important modules of the sample are the membrane and the double layer, and the modeling of these components is described in detail below.

#### 2.1 Membrane model

The original membrane model for cells [19] assumed transport of principal ions through the plasma membrane, which was controlled by respective equilibrium potentials for these ions, conductivities of the channels and membrane capacitance. Earlier, Fricke studied the influence of AC fields on red blood cells and showed that membranes exhibit a capacitive behavior [20]. The value for this capacitance (C_m_) was found to be 1 µF cm^−2^. Simulations of cell behavior in an electric field have been typically done using single or double-shell spherical models where testing of model parameters as well as cell manipulation such as dielectrophoresis or their membrane electroporation was conducted in the broad frequency range, field amplitude and pulse width [21], [22]. These models represent cells by using passive capacitive membranes of different relative permittivity connected to high value resistors and include low resistances of conductive electrolytes of cytosol and intercellular space [23]–[25]. Important membrane processes with associated transmembrane voltages as well as spatial and structural non-heterogeneity of cells and their suspension should be considered. Nonlinear cell behavior in electric fields can be represented by a nonlinear current dependent source I_m_(V_m_) as proposed by Gowrishankar and Weaver [26].



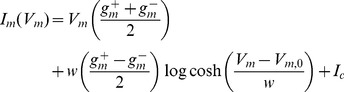
(1)where V_m_ is the voltage across the membrane, g^+^_m_ and g^−^_m_ are the maximum and minimum membrane conductances at very large and very small V_m_, respectively, w is the width of the sigmoid transition and I_c_ is a constant. Thus employing a voltage-controlled current source simulated the necessary behavior of the membrane.

**Figure 3:**
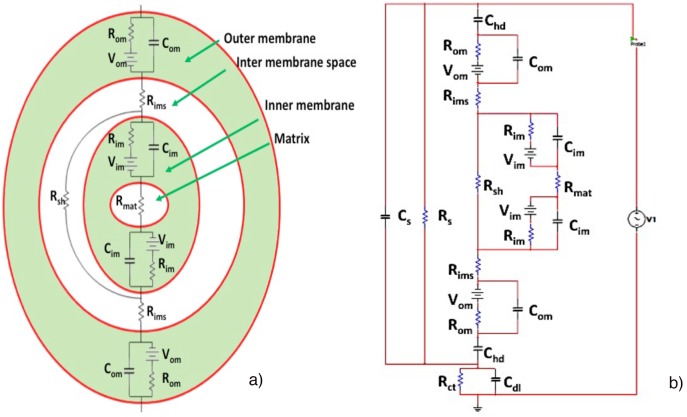
Equivalent circuit model developed for mitochondria (a). Note that this figure is not to scale. Complete equivalent circuit for the experimental setup is shown in (b).

In mitochondria, special interest is focused on changes of protomotive force i.e. membrane potential ΔΨ_m_ and ΔpH both in physiological conditions as well as under oxidative stress and various dysfunction processes. Equivalent circuits to be used to model mitochondria would have detailed electrical representation of important processes in outer and inner membranes [27]. A simpler version would include a battery with capacitances and resistances [28] in the inner membrane, and a proton circuit [7], [29] with a Zener diode [30]. Here, we will use a simplified model compared to [26]. We will implement the concept of voltage sources at the membranes connected with the high resistance and capacitance of the lipid membrane structures, which are nonconductive layers (Fig. 3a). Assuming constant values of the sources, corresponding to the optimal operation condition for healthy mitochondria we will test the influence of frequency on changes of mitochondria potential. Due to the difference in area, the impedance of the inner mitochondria membrane decreases much faster with frequency than the impedance on the outer membrane; that decreases the induced potential. The membrane resistance (R_m_) is very high and the current through this resistance represents the complex flow of ions across the membrane. The capacitive current reflects the change in the amount of charge separated by the membrane. Membranes have ion channels embedded in them, which control the flow of ions across them and are very selective. The ion channels and the ion gradients generated by them could be represented by current source in parallel with R_m_ and C_m_ as in the dependent source implementation [26]. With Thevenin equivalent, the ion channels control the charge transfer across the membrane, maintaining a specific charge gradient, V_m,0_. For typical cell membranes, with respect to the cell’s exterior, the potential difference between the intra and extra cellular environment is −70 mV.

#### 2.2 Electrical double layer model

The electrical double layer has a capacitive behavior. It can be modeled by a RC parallel circuit [31], [32] where the capacitor (C_dl_) represents the differential capacity of the double layer and the resistance (R_ct_) represents the Faradaic current on the electrode surface. We followed the Randles circuit to arrive at this equivalent circuit for the double layer.

With this information, we proceeded to model the mitochondria using an equivalent circuit model. The matrix can be represented by a resistor (R_mat_) and so can the intermembrane space (R_ims_). The outer and inner membranes are represented by a capacitor and resistor connected in parallel. Depicted in Figure 3a, it also includes a shunt resistive path (R_sh_) through the intermembrane space, which represents some cristae that stretch across the whole mitochondrion from one end to the other. To experimentally realize the equivalent circuit description, we added to the mitochondria equivalent circuit other components describing the sample suspension properties (R_s_ and C_s_ in parallel) and the ionic double layer parameters. This model is shown in Figure 3b.

In Figure 3b, R_om_ is the outer membrane resistance, C_om_ is the outer membrane capacitance, V_om_ is voltage across the outer membrane the current through the, R_im_ is the inner membrane resistance, C_im_ is the inner membrane capacitance, I_im_ is the current through the inner membrane and V_i_ is the applied voltage.

**Figure 4:**
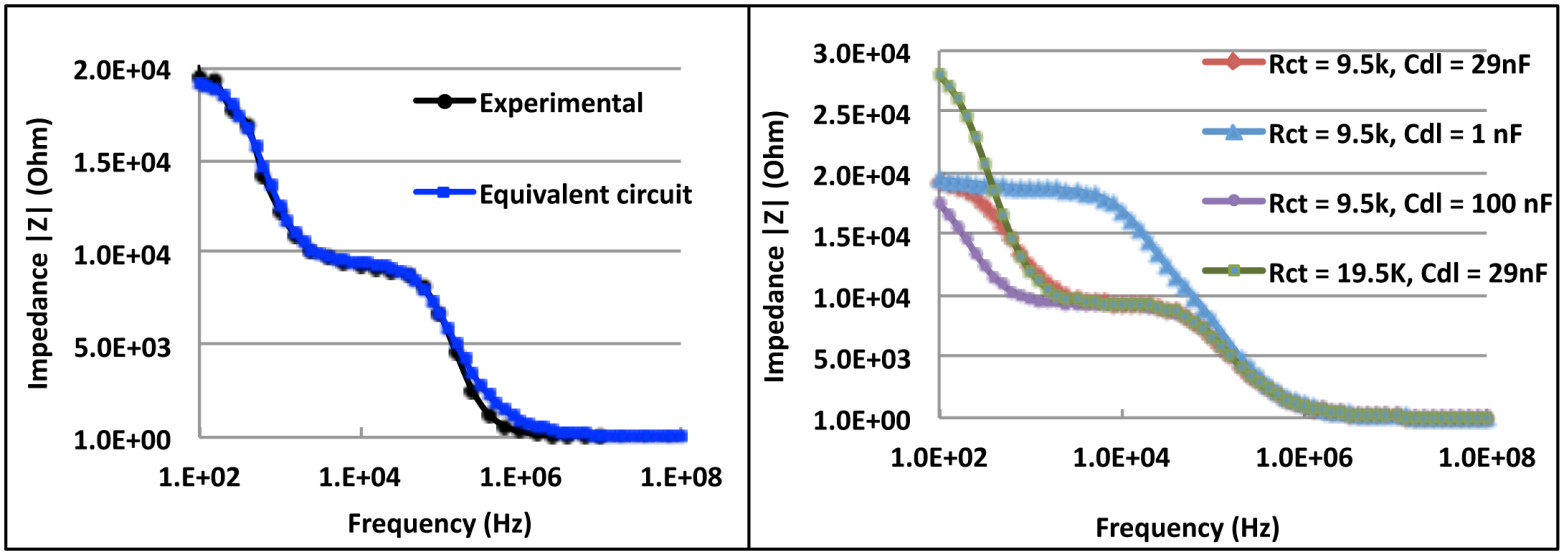
Plots of the impedance *vs.* frequency dependence for mitochondria sample calculated from the equivalent circuit (a) and by using different values of double layer capacitance C_dl_ in the equivalent circuit (b).

The resting potentials of the outer and inner membranes are known to be 70 mV and 150 mV, respectively. Outer membrane capacitance values were taken from the literature, [33] and inner membrane capacitance is known to be about 10 times larger than outer membrane capacitance because of the larger area. These values were used in the circuit, and by employing curve fitting, the values for the other elements were found. The experimental and fitting results are shown in Figure 4a. As can be observed, the experimental measurements fit closely with the calculated curve. Two dispersions are seen, one from the double layer and the other, membrane dispersions, at higher frequencies.

## Results and Discussion

### 1. Impedance spectroscopy measurements

Electrochemical Impedance Spectroscopy was done by connecting the electrode arrays to a Solartron impedance analyzer (SR 1260). Kramers-Kronig (K-K) Analysis was also performed to the spectral data. We used the analyzer built-in K-K compliant Voigt elements circuit model (-R-(RC)_m_-) to obtain the good fit of the model and thus obtained the electrochemical impedance spectrum from the analyzer using specialized software. Impedance spectroscopy was done on mitochondria samples as soon as they were extracted. Mitochondria take a spherical shape in vitro and hence we can assume that the sample consisted of shelled spheres. Detailed theoretical evaluation of such shelled spheres showed that the potential across the outer membrane has a Debye relaxation process when a sinusoidal field is applied. We identified such Debye relaxations from our measurements (Fig. 4a) and simulation (Fig. 4b). Because of polarization of bound charges on membrane surfaces and proteins, dispersions were expected, and at the frequency range of 1 KHz to 10 MHz used in these experiments, beta dispersions were observed.

At low frequencies, being capacitive in nature, the membrane exhibits high capacitive impedance, thereby rendering membranes highly impermeable to electric fields. As the frequency increases, membrane impedance decreases, and eventually the electric field could penetrate the membrane, leaving the membrane transparent at very high frequencies. Our measurements exhibit multiple relaxation processes arising from different phenomena. Here we see two relaxation processes. The first, at around 100 Hz, is from the capacitive behavior of the electric double layer at the electrode/electrolyte interface. The second relaxation process, at around 100 kHz, is from the mitochondrial membrane, as it too has a capacitive nature. The impedance values decrease with increasing frequency as the membranes charge and subsequently become transparent to the applied sinusoidal signal.

**Figure 5:**
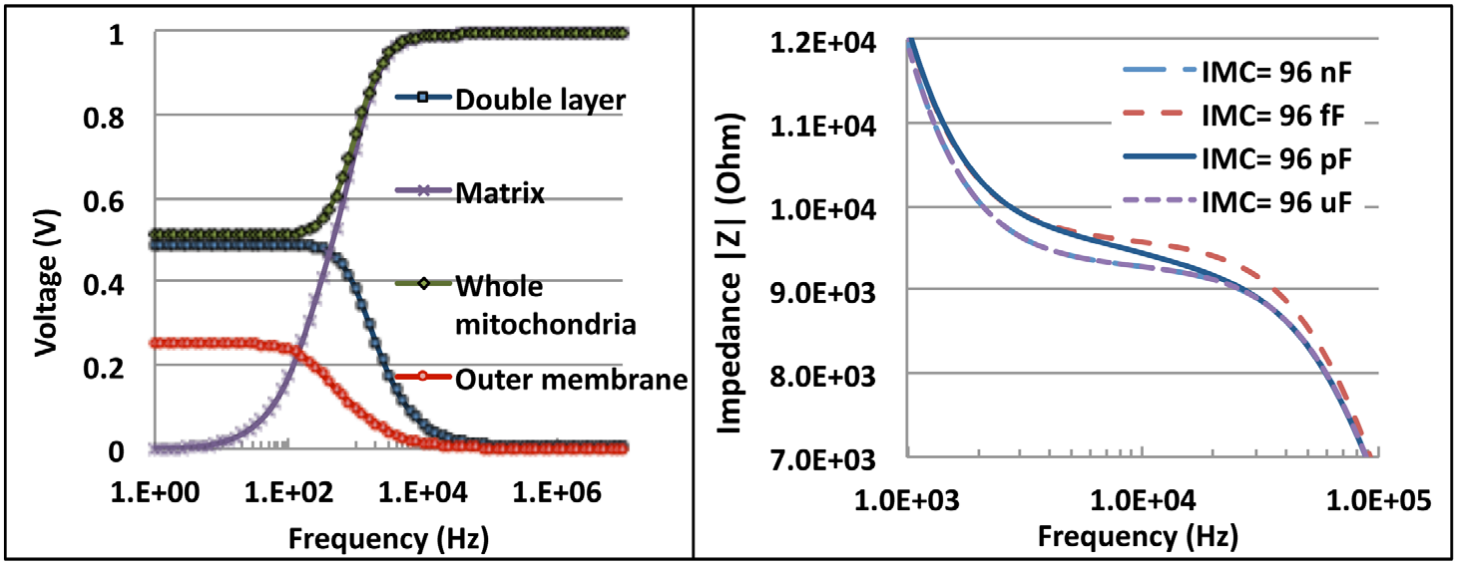
Frequency dependence plots of voltage at different regions of the mitochondria equivalent circuit (a) and of sample impedance influenced by the inner membrane capacitance (IMC) (b).

The voltage distribution across different parts of the circuit was studied to get a good understanding of the various processes going on and the interdependencies present. Voltages at some important points in the circuit are shown in Figure 5. From this graph (Fig. 5a) we see that initially, at low frequencies, the applied voltage (1 V pk-pk) was split between the double layer and the mitochondria sample (0.487 V across the double layer and 0.513 V across the mitochondria). Once the double layer relaxed, the entire voltage was applied across the mitochondria sample. The low frequency voltage was applied across the outer membrane. Only after the membranes relaxed could the voltage be applied to the matrix; this happened at 12.589 kHz. The 0.513 V applied across the mitochondria was split as 0.256 V across each part of the outer membrane. As the outer membrane reactance decreased, the voltage applied to the inner part of the mitochondria (inner membrane and matrix) steadily increased. The inner membrane voltage also increased till the capacitor relaxed; once this happened, the voltage across the matrix increased. From our simulations it appears that the outer membrane behaves as a low pass filter while the inner membrane behaves as a band-pass filter [24].

The charge gradient across the inner membrane is reflected in the capacitance across it (Fig. 5b); hence the capacitance C_im_ is a good parameter to study membrane potential. We see that C_im_ controls the mid-frequency impedance, and by increasing it by 1 nF, we got an impedance drop of 295 Ω (at 8 kHz). This correlates with the experimental results showing an increase in impedance when the charge stored was dissipated (reducing membrane capacitance) using FCCP. The simulation and experimental results show that the best frequency range to probe the inner mitochondrial membrane properties using electrical fields is between 100 Hz and 100 kHz; above that the membranes relax and their properties cannot be studied.

Frequency dispersion of dielectric properties has been recently simulated for cell suspensions [14] where model parameters such as membrane potential and thickness, mobility of charges at the membrane surface and conductivity of the media and intercellular fluid was derived to show match with experimentally observed permittivity dependence on frequency.

### 2. The role of uncouplers in mitochondria measurements

To study the response of the mitochondrial membrane potential changes, we added uncoupler FCCP and glucose to the mitochondrial suspension. It has been demonstrated [34], using optical sensing with fluorescent dyes such as TMRM that FCCP rapidly depolarizes the inner membrane by allowing protons to cross the membrane, thus simultaneously reducing membrane potential while increase the respiration rate. However, low concentrations of FCCP do not lead to complete membrane depolarization but, by increasing respiration, provides cardioprotection if administered prior to ischemia [35], [36]. Several effects related to ionic channels and induced currents include also major ions, such as sodium [37] and calcium [38], and affected pH values. As shown by Park et al. [39], FCCP also affects the plasma (cellular) membrane potential. The influence of this uncoupler on both mitochondrial and cellular membrane potentials, as well as induced ionic currents, depends both on extracellular and intracellular pH and FCCP concentration.

**Figure 6:**
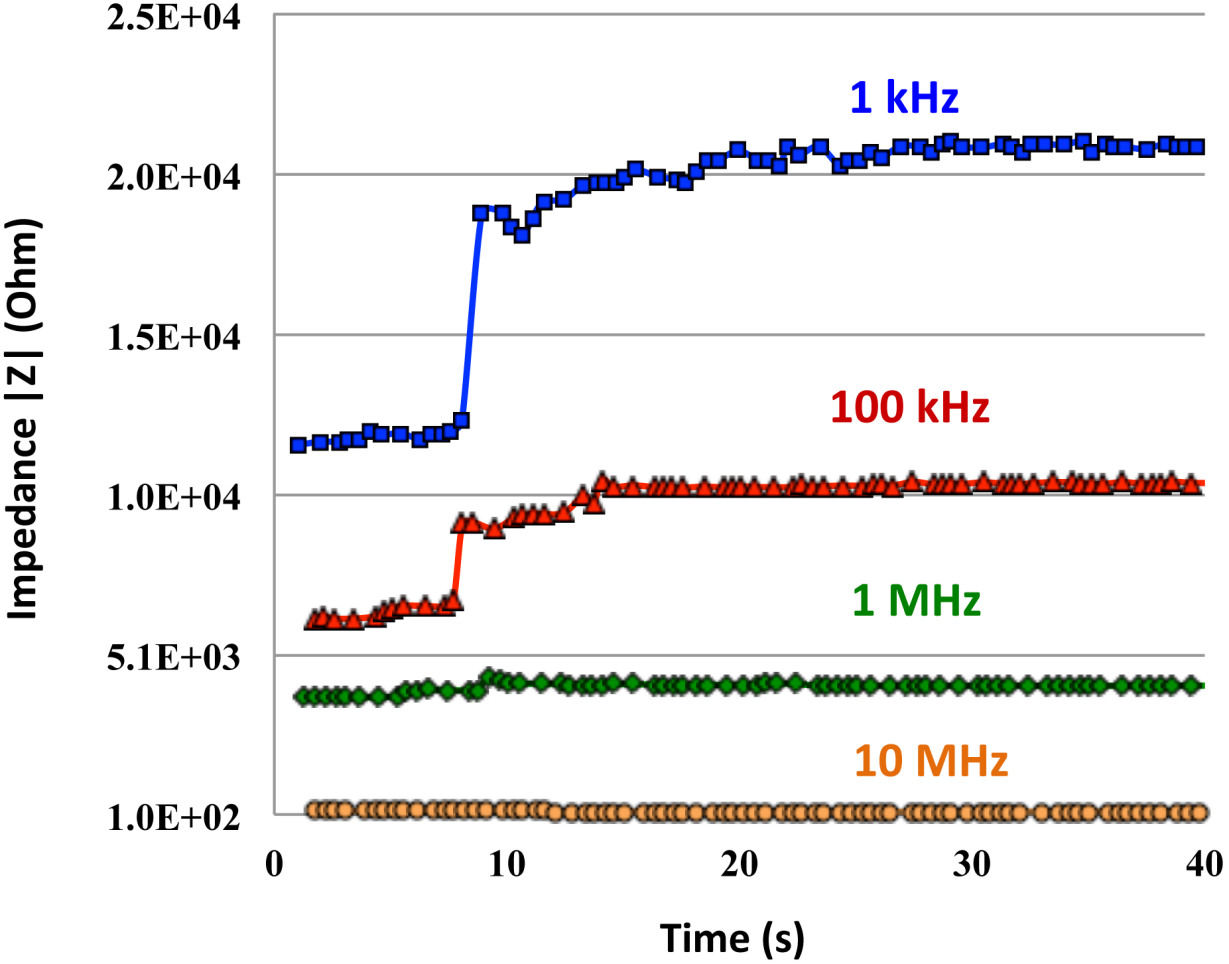
Dependence of measured impedance on time for four different frequencies is presented. Significant influence of FCCP addition (after 8 sec) can be seen for frequencies lower than 1 MHz.

Optical sensing is very powerful, especially in spatial selectivity but is prone to several experimental artifacts. Impedance measurements are also difficult for accurate interpretation. When FCCP was added to mitochondria (2 µl FCCP for about 0.75 ml mitochondrial sample), the impedance increased because of the reduction in membrane potential and dielectric response [14], with additional contributions by the outward current flow [40] across the membrane as protons rush inside the matrix. The impedance increased by 3.3 kΩ at 1 kHz for mesh electrodes, and by 9.6 kΩ for nonmesh electrodes. The experiment with FCCP was repeated at different frequencies, 1 kHz, 100 kHz, 1 MHz and 10 MHz. (Fig. 6). Control measurements using buffers without mitochondria were conducted to verify that the observed impedance changes are caused by FCCC acting as uncoupler not as a chemical modification of the solution.

Due to the differences in inner and outer membrane capacitances their sensitivity to frequency favors lower range of frequency measurements, but beyond electrode polarization. The largest observed change in impedance was at 1 kHz and decreased with frequency. The changes in impedance typically noted were up to 10 kΩ, 4.38 kΩ, 390 Ω, and no change for 1 kHz, 100 kHz, 1 MHz and 10 MHz, respectively. The impedance response was reduced at higher frequencies compared with low frequencies where the field had not yet penetrated the membranes (which can be observed in Fig. 4). Therefore, these measurements reflect the membrane response. As frequency increases, the membrane is transparent to the applied signal, and thus its response is negligible. This happens at around 1 MHz, thus we see only very small changes in impedance at frequencies above this. We are also safely far away from double layer effects at 100 kHz; thus these data are reliable, and represent only membrane properties.

**Figure 7:**
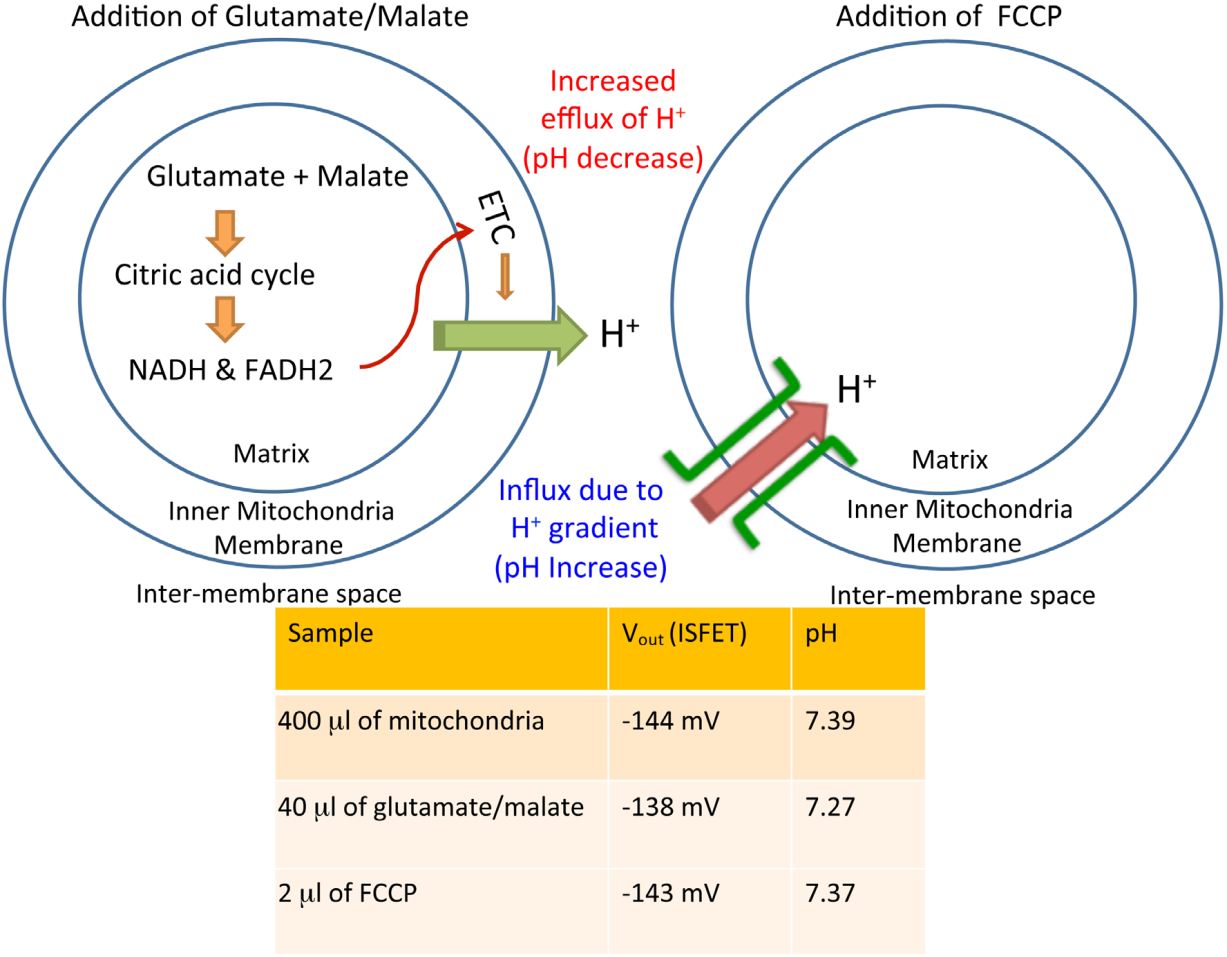
Schematic description of the influence of glutamate/malate and FCCP on external pH is depicted.

For corresponding ionic studies, initially 800 μl of mitochondria was placed in a chamber, and output voltage in the ISFET was noted for the setup as −144 mV, which could be translated to the pH value of 7.39 using the calibration curve. After 40 μl of glutamate/malate solution was added to the mitochondrial sample, the output voltage was observed to be −138 mV, suggesting a fall in pH to 7.27. This fall in pH is related to the efflux of hydrogen ions during the respiration process induced when the oxidation-substrate (malate-glutamate mixture) was introduced into the mitochondrial suspension (Fig. 7).

Detailed experiments with ionophores FCCP and valinomycin resulted both [41] in the decrease of ΔΨ_m_ and change in pH, which then affected ROS generation. Addition of FCCP, known for its acidification of the matrix, further increased the pH to 7.37 because of opening of proton pores in the mitochondrial membranes. All the chemical solutions used here were pH-adjusted and control experiments were repeated with pH-adjusted buffers to confirm that the results were not from the acid–base reactions of the chemicals in the aqueous phase environments. Hence we observed a substantial response in pH, correlating well with expected ionic response. However, measurements of impedance response to FCCP show higher sensitivity than that of pH.

## Conclusions

We have developed an integrated sensor to study various parameters associated with mitochondrial functions. Such an approach can aid in the *in vitro* characterization of mitochondrial function or dysfunction, which has been implicated in many major noninfectious diseases of the twenty-first century. Impedance spectroscopy was studied as a tool for probing changes in mitochondrial membrane potential and shows great promise for development as a stand-alone measurement technique for membrane potential studies. Parallel ionic measurements were taken, which are necessary in such a system, where the ionic pathways play a prominent role in controlling the functionality and efficiency of the mitochondrial system. The effects of uncouplers on the impedance of a mitochondrial suspension were studied, showing an understandable and promising correlation between impedance and membrane potential.

We have also developed an equivalent circuit model for the mitochondrial suspension, which will enable an easier and straightforward extraction of the actual membrane potential numbers from the sample impedance. A similar trend was observed with impedance changes seen with the changing inner membrane capacitance in the equivalent circuit and the experimental results of impedance change with FCCP. Thus we have developed a novel and unique method of probing mitochondrial membrane properties by studying their dielectric behavior. The technique, when further developed, can be used for whole cell impedance studies. Quantitative interpretation will require a more advanced model, e.g., [42], which incorporates the mitochondria into the cell and also most likely uses a different frequency window. The tool discussed here is simple in construction and less noisy than cytometry techniques. One can study dynamic response without the use of fluorescent labels, which could alter mitochondrial behavior; it also is very versatile in design, as one can study different mitochondrial responses simply by modifying the equivalent circuit.
